# Diagnostics Now in PubMed and PubMed Central

**DOI:** 10.3390/diagnostics6010012

**Published:** 2016-02-23

**Authors:** Andreas Kjaer

**Affiliations:** Editor-in-Chief of Diagnostics. Department of Clinical Physiology, Nuclear Medicine & PET and Cluster for Molecular Imaging, Rigshospitalet and University of Copenhagen, Blegdamsvej 9, DK-2100 Copenhagen, Denmark; akjaer@sund.ku.dk; Tel.: +45-3545-4216

It is with great pleasure and proud that we can announce that *Diagnostics* is now indexed and searchable in *PubMed*. Further, the full-text versions of the papers are archived in *PubMed Central*. Since the launch of our journal in 2011, this has been a major goal for the editors and the publisher. With coverage in both the index and full text repository of the *National Library of Medicine (NLM)* articles published in *Diagnostics* will be more visible and hopefully reach more readers. The coverage will be from the first issue, so, additionally, papers published prior to coverage will become accessible via the databases.

The journal is currently developing in a most positive way with an increasing number of submissions, a total of 60 in 2015. A total of 37 papers were published in 2015 of which 43% were full-length research articles, 35% reviews and the remaining case reports or interesting images. Interesting images was introduced in 2015 [1] and we are receiving an increasing number of submissions within this category. Submissions are received from a variety of countries worldwide.

The total number of downloads of *Diagnostics* papers from our website was 37,377 in 2015, an increase of 139% compared to 2014. Citations are also increasing steadily with many papers being highly cited. Information on most cited papers may be found on the webpage of *Diagnostics*.

With the continued support from the scientific community through submissions of high quality scientific papers and the extraordinary work performed by the editorial board, office staff and the publisher, I have no doubt that 2016 will be another successful year for our journal.
